# Changing Knowledge, Principles, and Technology in Contemporary Clinical Audiological Practice: A Narrative Review

**DOI:** 10.3390/jcm13154538

**Published:** 2024-08-02

**Authors:** Sophie Brice, Justin Zakis, Helen Almond

**Affiliations:** 1 Australian Institute of Health Service Management, COBE, University of Tasmania, Sandy Bay, Hobart, TAS 7001, Australia; 2 Institute of Health Management, 185-187 Boundary Road, North Melbourne, VIC 3051, Australia; 3 National Acoustic Laboratories, Level 4, 16 University Avenue, Macquarie University, NSW 2109, Australia

**Keywords:** teleaudiology, clinical audiology, remote care, technology, translational science, clinical audiology

## Abstract

The field of audiology as a collection of auditory science knowledge, research, and clinical methods, technologies, and practices has seen great changes. A deeper understanding of psychological, cognitive, and behavioural interactions has led to a growing range of variables of interest to measure and track in diagnostic and rehabilitative processes. Technology-led changes to clinical practices, including teleaudiology, have heralded a call to action in order to recognise the role and impact of autonomy and agency on clinical practice, engagement, and outcomes. Advances in and new information on loudness models, tinnitus, psychoacoustics, deep neural networks, machine learning, predictive and adaptive algorithms, and PREMs/PROMs have enabled innovations in technology to revolutionise clinical principles and practices for the following: (i) assessment, (ii) fitting and programming of hearing devices, and (iii) rehabilitation. This narrative review will consider how the rise of teleaudiology as a growing and increasingly fundamental element of contemporary adult audiological practice has affected the principles and practices of audiology based on a new era of knowledge and capability. What areas of knowledge have grown? How has new knowledge shifted the priorities in clinical audiology? What technological innovations have been combined with these to change clinical practices? Above all, where is hearing loss now consequently positioned in its journey as a field of health and medicine?

## 1. Introduction

The origin of audiology as a clinical area of expertise is known to be the formative work by Carl Bunch in the 1940s [1]. At this time, the research question driving what would become audiology was to ask how physical limitations impacted a person’s functional capacity, which the area of psychology governed, i.e., ‘psychophysics’ was the parent domain of audiology. The Second World War created a rise in hearing impairments among returning army veterans, which enabled work on the improvement of testing methodologies for hearing, along with the development of devices—most commonly, hearing aids—to compensate for the identified deficit accordingly. Treating hearing loss was, thus, grounded in the physical components of hearing and the loss thereof.

The objective of hearing aids is to amplify sound to compensate for the diagnosed loss of functional hearing. This requires being able to measure the degree of loss and prescribe hearing aids that have been personalised for the diagnosed loss. The clinical process, therefore, consists of diagnosis, treatment, and follow-up to check progress has been made (see Table 1). In other words, the steps are the assessment of hearing, fitting of a hearing aid, and rehabilitation to ensure the hearing aid provides an improvement in functional hearing. These three key clinical steps remain fundamental in audiological practice today.

There have been many innovations impacting clinical practice since the 1940s. The objectives of the core clinical stages remain consistent, though the methodologies available have allowed greater choice and versatility in how the core objectives are addressed. A recent shift in audiological methods has been heralded by the digital age, enabling almost every step of clinical audiological practice to be carried out remotely. The acceptance and adoption of remote-technology-enabled practices are evident in the production of clinical guidelines that acknowledge and support the modern practice referred to as teleaudiology [3]. Teleaudiology is a term used to refer to a delivery mode of an audiological service [3] and is gaining traction as an enabler rather than a barrier to audiological practices that are otherwise limited by the confines of a traditional clinical design.

The automation of audiometry was the first great revolution in audiology, allowing the clinician to improve the quality and range of reliable data used to diagnose deficits in hearing. At the time, change in the clinical methodology was recognised as a triumph for clinicians, since they could apply their time more effectively for the analysis and interpretation they were trained and qualified for, rather than manual tasks that were no longer necessary and occupied so much of their valuable time [6]. The technological advancement afforded by the audiometer impacted the clinician’s role by enabling more data to be collected to inform the clinical decision-making process and, consequently, changing the logistical demands on the clinician for the better. Technology-enabled automation is at the root of modern changes in audiological practices, as it further changes the logistical demands on the clinician and enables more forms of data to be collected. This adds even more information to assist the clinician in determining how they may assess and interpret hearing performance, fit a prescribed hearing aid, and manage the rehabilitation and progress of the people being treated.

A key problem in adult audiological practice, however, remains in enabling the person with hearing loss to be willing to seek, adopt, and adhere to the high-quality help that is available today. It appears all of the technological advancements aiding clinicians so far face different challenges, which involve enabling the help that clinicians give to be utilised to its greatest potential by the person at the centre of it all—the person with hearing loss. There is an arguably poetic element in the fact that psychology was initially the parent domain of audiology as a new field of study and remains an integral part of supporting a person’s choice to seek, adopt, and adhere to the clinical help today’s audiology professionals can now offer. A key feature of the technological advancements that can be explored in contemporary audiological practices is in utilising online capabilities to enable the inclusion of the person being treated as a result of logistical opportunities in new methods.

Partial, equal, or full control of a task by the person being treated—no longer only the clinician—is now possible for most if not all clinical duties. The idea of affording a person agency or autonomy in their own care, which is commonly referred to as person-centred care [7], is as old as the field of audiology. So, it is with no great irony that the modern technology-enabled merger of these two fields has an opportunity to enhance contemporary audiological practice. The problem of low rates of adoption and adherence in audiology has been well documented, and recommendations to explore how the autonomy enabled by teleaudiology could involve the practice of person-centred care to benefit audiological practices have been presented [8,9]. In other words, engaging a person in their care as a result of utilising teleaudiology to promote autonomy may be an opportunity to address the barriers of low adoption and adherence in audiological care.

Taken together, could the utilisation of recent advancements in audiological knowledge and capabilities help leverage consumer engagement in the battle towards improving adoption and adherence in audiology? Simply put, with more knowledge of the science and psychology of hearing loss, can the success of the field of audiology be improved with the opportunities afforded by new or newly informed methods and practices?

The last five years have seen major revisions to clinical guidelines in adult-focused audiology services in the USA and Australia, which have been marked by differing approaches to incorporating teleaudiology (see Table 1). The pace of innovation is unpredictable, and it is hard to plan for what other considerations will be impacted. While there is a growing body of literature on the methods and practices of teleaudiology, it largely follows methods that evolved from traditional clinic-based audiological practice [10]. Advances in scientific knowledge and insights, however, could help bridge the gap between using new tools to support old methods and the potential to identify new purposes or processes to inform all audiological practice [11]. The rationale for this narrative review is to consider the potential opportunities that teleaudiology may bring to audiological practice in adult services from the perspective of current scientific developments. This narrative review, therefore, aims to unpack advances in audiological science across the body of knowledge and consider how these impact clinical practices in audiology in the context of teleaudiology.

## 2. Review

Advances and new information on loudness models, tinnitus, psychoacoustics, deep neural networks, machine learning (ML), predictive and adaptive algorithms, and patient-reported experience/outcome measures (PREMs/PROMs) have enabled innovations in technology to revolutionise clinical principles and practices for (i) assessment, (ii) the fitting and programming of hearing devices, and (iii) rehabilitation. This narrative review will consider key advancements in knowledge and technology and their potential to create positive change together with teleaudiology, as well as the key barriers to success, i.e., adoption and adherence to wearing of hearing aids, in contemporary audiological practice.

### 2.1. Assessment

The first clinical stage of audiological practice is to assess the patient’s hearing ability and/or loss. The measurement of hearing thresholds using pure tones is the oldest and most relied-upon approach for gauging the degree of hearing loss. Technology has enabled pure-tone audiometry to be conducted from potentially any device with a speaker (even over the phone), so people with hearing loss can manage the test independently if they wish [12,13,14]. Advancements in the knowledge of auditory science have shown that pure tones alone are not sufficient to indicate the integrity of more complex aspects of how the auditory system functions, either normally or in the presence of hearing loss. The array of current clinical testing methods includes various technologies for sound reproduction and recording using specialised speakers and microphones in various formats, as well as technologies for recording brain wave activities using specialised electrodes. The three main levels of information being gathered can be summarised as follows:

- What does the person consciously detect or recognise?
- What is neurologically detected regardless of conscious cognitive input?
- What impact does the physical pathway of sound from the ear canal to the site of conversion into a neurological signal have on the sound that is detected?

Data collected with current clinical testing methods for hearing provide a profile of functional hearing and can indicate the nature of the loss of hearing. However, mainstream clinical responses to hearing loss are to prescribe a hearing aid and use tone-based data to program the hearing aid accordingly. The remaining clinical data collected assist with counselling for likely benefits and expectations where needed, in addition to indicating where further medical assessment may be warranted.

Pure-tone thresholds are used with a prescriptive procedure to personalise the amplification settings of the hearing device for a person with hearing loss. Common prescriptions do not incorporate individual data on speech discrimination ability [15], context dependence in noise-filtering ability [16], individual variations in loudness growth patterns, or abnormal auditory amplification mechanisms [17] of a person’s auditory processing system (see Table 2, Table 3 and Table 4). Incorporating data on some or all of these supra-threshold factors could enable more personalised hearing-aid fittings that are more satisfactory to people with hearing loss and could lead to a reduction in the rejection of hearing aids. However, collecting the amount of data needed to achieve such an objective may require more time than is clinically feasible, with current service provision models using a smaller array of testing methods. The increasing variety of clinically validated tests that can be conducted via teleaudiology for remote testing [18] or for a person to conduct independently [19], with the clinician remotely receiving the data, is an opportunity to consider in order to improve clinical efficiency. Similarly, over 60 years ago, audiological practices benefited from the technology-enabled automation of testing methodologies, which was praised for allowing highly trained clinicians to use less time for manual tasks and prioritise the more valuable part of their clinical training—analysis and interpretation for clinical decision-making in the selection and delivery of treatments for hearing loss [6,20].

Typical processes for assessing and fitting hearing devices may be a clinically driven service, but the choice to adopt and adhere to wearing hearing aids is ultimately not a clinically controlled event. Subjective and behavioural components of adopting and adhering to hearing aids are made clear in the instances of rejection of hearing aids, even in the presence of objective evidence of their benefits [21,22]. The key behaviour needed for hearing aids to be successfully used in the management of hearing loss is rooted in the person’s autonomy in choosing to do so. Autonomy has been proposed as an enabler of adoption and adherence to wearing hearing aids, with teleaudiology being a fundamental complement to the promotion of autonomy. It is, therefore, of interest to consider if autonomy enabled by teleaudiology in the assessment phase may provide more help than hindrance for clinical success in diagnosing and treating hearing loss.

Teleaudiology has been a valuable tool for increasing access to and provision of audiology services, and online communities for testing, online clinical appointments, and rehabilitative or follow-up appointments have increasingly been utilised. Benefits are possible for remote communities, individuals with limited mobility, and those whose lifestyles are not amenable to typical clinic visitations. A retrospective study of commercial data over a 12-month period of operation in 2018 that analysed service choices relative to the proximity to a clinic revealed that, while close proximity to a clinic may influence the choice of service that a person makes (60% clinic services vs. 36% online services for those living with 50 km of a clinic), there will always be consumers who choose online services despite being close to a clinic, and some consumers will always choose to travel large distances despite the option of online service counterparts [23]. Over the last 10 years, there has been growth in the number of assessment tools available for consumers to complete independently online [19]. The variety of tests available allows the consumer to be informed and provides opportunities for clinicians to monitor individuals who may require recommendations to proceed further and to monitor changes over time, such as when a sudden change in hearing may require urgent medical attention to prevent permanent loss [24]. The first key advantage of the online accessibility of audiology assessment tools is, thus, the increase in their potential reach, targeting those who specifically cannot or will not attend a clinic. The second potential advantage that may help combat challenges of low adoption and adherence in audiological practice is the increase in engagement with individuals who may otherwise proceed to reject the hearing aids and/or clinical services that they need. There are currently limited data to support this point [8]. An audiology service model providing a blend of clinic-based and online services in Australia was used to analyse the relationship between the use of self-assessment tools and the likelihood of adopting hearing aids online over a three-year period [25]. The adoption of hearing aids via teleaudiology was significantly higher when the help-seeking phase was led by self-assessment tool use than any other method of contact or service model chosen, indicating a vital need to consider the behavioural impact on hearing aid adoption when assessments are made available through teleaudiology. Another blended model from South Africa demonstrated that the online pathway of access to assessment tools via teleaudiology could only be associated with demographic factors and the number of services required rather than the mode of service [26]. A new era of automated assessment methods, including utilisation of teleaudiology to redistribute the workload of certain tasks, may allow clinicians to prioritise their clinical work on tasks that provide more value to the success of services for diagnosing and treating hearing loss.

The capacity for technology to automate minor steps in assessment methods can also be demonstrated by the introduction of ML to the standard practices of audiometry [27]. Logistically, automation of the choice and delivery of tones across the range of frequencies available allows clinical software or an app to lead the data collection process with the person being tested. The ultimate advantage is that the process can be completed without direct supervision of a clinician, creating flexibility for self-testing and asynchronous data collection and, thus, providing clinicians with a choice to plan assessments and care as suits the clinical and personal needs of all involved. An exploratory study of the possibilities of combining expert judgement with technological power was recently reported by Dou et al. (2024) using over 12,000 audiograms and 3 experienced clinicians to create a deep learning framework for assisting in interpreting and clinical decision-making for a range of classifications of degrees of hearing loss, as well as types and configurations thereof [28]. Zaar and Carney (2022) proposed utilisation of deep neural networks and machine learning to manage and process the amounts of data for the provision of extensively personalised auditory profiles to support such an objective [29]. Use of deep neural networks has been in development to improve noise-filtering algorithms for over a decade [30], bringing improvements to the speech recognition abilities of those using modern hearing aids [31]. The next step is to bring individual measurements of auditory processing activity together with such networks to refine and personalise the sound processing in hearing aids and further improve the outcomes for wearers [32,33]. Potential for the complete personalisation of hearing-aid fittings can be achieved when clinical data and the wealth of current scientific and individual insights that are now available are combined to impact clinical decision-making in the treatment and management of hearing loss.

Therefore, the science on the generation, transmission, conduction, sensing, and processing of sound, from an acoustic (independent of the person receiving the sound) to an auditory (dependent on the person receiving the sound) phase, are of fundamental importance. Table 2 shows an overview starting with the environmental stage, where soundwaves are generated from one or more external sources, such as speech emitted from a person’s mouth. These soundwaves travel to the recipient’s ears via direct and indirect (acoustic reflections) environmental transmission paths, which modify the level, spectrum, and phase characteristics of the sound in different ways. The conductive stage begins where sound from different sources and transmission paths travels through the pinna (external outer ear), which modifies the characteristics of sound based on the direction of arrival. Soundwaves then travel through the ear canal and cause movement of the ear drum. In the middle ear, eardrum movement is transferred to the oval window (or membrane) of the cochlea via a chain of three miniature bones. In the inner ear, movement of the oval window is transmitted through fluid inside the cochlea, causing movement of the basilar membrane. Different locations on the basilar membrane have maximal movement in response to different tones (frequencies). In the sensory stage, basilar membrane movement causes inner/outer hair cells (IHCs/OHCs) at the same location to bend and activate receptors, which transduce movement into electrical signals. In the neural stage, electrical signals from a hair cell are transferred to a nerve cell, and then converge into neurons shared by multiple hair cells. This stage initiates a neural pathway that proceeds from the inner ear (cochlea) to the uppermost cortex of the brain. In the auditory processing stage, many nerves leaving the cochlea meet at sequential junctions between the cochlea and auditory cortex of the brain, pooling their signalling patterns in complex and specific patterns, effectively acting as a very complicated relay from the cochlea to the cortex. As the relay of signalling proceeds in a hierarchical fashion, there are binaural transverse connections, adding further complexity [34].

Technology-enabled improvements in hardware and software supporting the automation of clinical methods can also be seen in the adaptation of Oto-Acoustic Emission (OAE)-based assessments. Technology innovations can occur from changes in function and purpose combined with deeper understanding of systems and processes. Within the auditory system, OHCs play the key mechanical role of amplifying the movement that instigates sensory transduction in the OHCs as well as the nearby IHCs. The mechanical motion, however, can also lead to propagation of waves travelling in the reverse of the original sound pathway, resulting in internally generated movement of the ear drum that creates a soundwave in the ear canal, known as an Oto-Acoustic Emission (OAE). The dependence of OAEs on predominantly OHC functionality allows the simplistic application of ear-canal-based microphones in potentially low-tech setups, such as affordable headphones [35]. Smartphone apps and accessible equipment now allow more testing methods to be delivered remotely, affordably, and/or asynchronously to meet the needs of clinicians and people, especially where remote assistance may not be feasible [35]. The technology of these methods allows teleaudiology to be a practical choice for planning and delivering clinical services in various settings.

The OHCs are implicated in multiple testing methods due to the complexity of various parts of the auditory processing ecosystem, rather than due to complexity in their own cellular functioning. Sound travelling from an external source, into the auditory system, and onwards for processing will incur the passive movements of the basilar membrane that induce signalling via the OHC and IHC, as well as a degree of active mechanical response from the OHC to exacerbate the movement. The ensuing transduction of the mechanical movement of the OHC to an electrical signal in the synapse-linked neurons is a bottom-up form of signalling, i.e., afferent. OHCs, however, can also be ‘asked’ to mechanically respond to the passive movements of the basilar membrane, or deliberately not, from other synapse-linked neurons that provide signalling to them (efferent), as part of ‘top-down signalling’ (further explored in Table 4). The functional value of top-down signalling to the OHC is to selectively amplify or dampen sounds. To do so, the integrity of the neuron, the synapse, and the OHC itself are all implicated. Lack of amplification is implicated in loss of sensory signalling; however, loss of top-down signalling is also implicated in the loss of ability to dampen any detected ‘noise’, meaning that hearing loss is a loss of selectivity as much as it is a loss of ‘hearing’. Pure-tone threshold testing, OAE-based testing, as well as speech-based testing are thus all potentially able to reveal different facets of OHC function, integrity, and the broader processes of the auditory system. All three types of testing are common in clinical practice and available with teleaudiology [13,14,35].

The integrity of OHCs, their associated synapses, and the neighbouring neurons shape the measurement of hearing performance at the threshold level, conversational level, and loud levels of speech. Speech recognition performance in quiet situations can, in some instances, be above average due to reduced tone-specific dampening action by the OHCs, whereas more challenging listening situations with the presence of noise then reveal a more greatly compromised hearing performance with the same pathophysiology [16,36]. Remote pure-tone testing, speech testing, and speech-in-noise testing are all established methods that can be used to support the seeking of help and remote fitting of hearing aids. Expanding the array of tests to include varying comparative loudness levels within a test or using linked tests may produce more useful data for clinical decision-making. Pairing microphone- and speaker-based data on a smart device may add valid real-world measurements of the environment to complement hearing performance and/or ratings that align more closely with a person’s daily experience [37]. Together, teleaudiology-based data collection can occur using a variety of methods, either before a person has been fitted with hearing devices or conducted as part of the care planning after the fitting of hearing devices.

### 2.2. Fitting and Programming of Devices

#### 2.2.1. Hearing Aids

Hearing loss is predominantly treated through the fitting of hearing aids for daily use to compensate for diagnosed loss of functional hearing ability. Audiometric data collected in the assessment stage are the key information that are clinically used to prescribe the initial amplification settings. This may be followed by further adjustment and programming of the hearing aids to ensure optimal hearing ability for the user. The objective benefits of hearing aids, however, may not suffice to ensure that a person adopts or adheres to the clinically prescribed use thereof, with conflicting data from those who do reject their hearing aids, indicating that more than clinically valid benefit is needed to ensure success outside of the clinic [21,22]. It has been observed that at least 98% of people fitted with hearing aids will experience at least one issue once they begin to wear hearing devices in their daily lives, which is further complicated by whether they communicate their issues to the clinician. This highlights the complex nature of how success is assessed by the people wearing hearing aids [38]. Objective clinic-based measurement of benefits and the subjective person-derived decision as to whether a patient will continue to wear their hearing aids are, thus, key challenges in current audiological practices.

Recent research has provided a wealth of information and new technologies that could be vital in shaping new directions for how to address the problem of hearing aid rejection. The complexity of sound detection and processing from the ear canal to the highest level of the auditory cortex and into other domains of our brains now shows us many reasons why assessing hearing with pure tones at threshold levels does not capture many of the other indicators of potential performance with hearing aids (see Table 2 and Table 4). Current knowledge reveals a myriad of scientific insights pertaining to the many factors that lead to individual differences that a person may experience in the integrity and functional ability of their auditory systems, which shape their personal—and diminishingly subjective—experience. If data could be collected using modern clinical methods and analysed with newly informed interpretations and evidence as a standard clinical data management practice, could improvements be made to the clinical management of hearing loss [39]?

**Table 3:** Case examples of the potential application of personalisation and clinical profiling.

Audio Stages Implicated	Current Scientific Insights	Clinical Implications	Teleaudiology Applications
Sensory Neural Auditory Processing	Despite IHCs providing the primary source of signalling to the neurons, significant loss of IHCs does not cause a shift in the detectable threshold of tone-specific hearing. Loss of OHCs, however, need not be significant to lead to a detectable shift in thresholds [40].	The threshold of hearing is not reflective of the diffractive effect on hearing performance in increasingly challenging environments, such as those with loud or competing noise.	Comparing test stimuli to provide a comprehensive portrayal of auditory factors could support gauging where the optimal benefit with fitting should be for individuals.
Sensory Neural	Noise-induced damage primarily builds up at the synapse connecting the hair cell to the nerves [41]. Age-related loss is considered to have a cumulative effect on the integrity and function of synapses and their respective nerves across the auditory pathway over time. Short-term noise damage to a hair cell can be repaired at the cost of a long-term impact on the health of the associated synapse and nerves [40,42].	Prompt testing and tracking of threshold shifts and recoveries are key in triaging the need for further medical intervention, and they provide a comprehensive time-sensitive profile of auditory health status over one’s lifespan.	Prompt data capture and responsiveness to acute/short-term changes in hearing can be provided. Teleaudiology-based capture can support the triggering of medical and clinical processes in local services.

The current approach to programming hearing aids during the initial fitting involves the selection of one of a small range of prescriptive procedures [43,44]. The two most widely used prescriptive procedures, NAL-NL2 and DSLv5, have been in clinical use for at least one decade [43,44]. However, while amplification can make speech as loud as normal (if desired), speech understanding may not be normal due to distortions in neural speech processing caused by hearing loss [45,46]. The effect of these distortions on speech understanding increases with the duration of hearing loss, although the process of degradation can be slowed (but not reversed) with the use of hearing aids [47,48]. In other words, the use of hearing devices serves to slow the progression of neural processing distortions rather than to stop or reverse it. The cornerstone of contemporary knowledge in auditory science lies in understanding why there is such great variation in the experiences that people have with hearing aids. As an example, a recent study showed that with amplification based on NAL-NL2 or DSLv5, speech understanding in noise was comparable when averaged across participants with different types of moderate hearing loss (conductive, sensorineural, or mixed) [49]. However, the authors noted that individual needs and preferences should be considered when selecting a prescription, since NAL-NL2 and DSLv5 can prescribe different gain–frequency responses for the same audiogram [50]. This reflects how these prescriptions were derived with different goals, research data, and/or perceptual models. Currently, there is an opportunity to improve prescriptions with new research data and/or improved perceptual models. Furthermore, modern machine learning algorithms can be utilised on much larger sets of more diverse clinical assessments and outcomes, which could provide a more complete profile of functional hearing to further individualise fittings. This could result in more satisfactory amplification settings that require less fine-tuning for individual needs. An insufficient benefit from hearing aids remains a main contributing factor behind the negative experiences that lead to the high rate of rejection [21,51].

Clinical decision-making processes of selecting an appropriate hearing aid, determining the amplification prescription, and further programming have been shaped by methods compatible with teleaudiology, which now allow a clinician to conduct the fitting and programming remotely [10,52]. Once an amplification prescription has been applied to a hearing aid with a person’s hearing data that were collected during an assessment, there is a common and almost ubiquitous step of refining the prescribed amplification for an individual’s needs and preferences [53]. Real personalisation of hearing aids is, thus, so far arguably as manually derived as it is automated via clinical hearing assessment data. Further refinement of the clinician’s workload could occur by automating the selection and application of hearing aid programming options. Databases of clinical assessments, fittings, and, crucially, preferred post-fitting adjustments to hearing aid settings are an exciting opportunity to close the gap between the quality and quantity of clinical data and clinical decision-making and efficiency [54]. Reducing the time a clinician spends personalising a hearing aid fitting as a result of individual differences in auditory processing abilities, the fitting process could become even more amenable to teleaudiology-based delivery, where increased access, reach, and clinical success rates can potentially be achieved. The automation on which teleaudiology has capitalised could positively reshape how clinicians spend their time, ultimately aiming to maximise the application of their knowledge, skills, and training to increase success in diagnosing hearing abilities in more detail and providing treatments that avoid rejection. Enabling consumers to be more active participants in their care also has great potential to improve adoption, adherence, satisfaction, and outcomes [8,55]. Together, the clinician and consumer have much at stake with the use of teleaudiology to increase the scope and capability of the modern audiology clinician and to improve consumer engagement and experience.

The validation of a fitting is an important step in reducing the risk of rejecting a device. A key goal of validation, however, should ideally point to the device user’s sense of how well the device will serve them, demonstrating to them that the device does indeed provide benefits. Within the context of a clinic, the real-world ecological challenges that a person experiences with their hearing may not be effectively captured, reflected, or recounted [56]. Speech-based tests represent a real-world measure of benefit from hearing aids and can be used to provide an indication of the benefit that a person should expect from their hearing aid [57]. Classifying the amount of benefit that a person can expect from a hearing device is limited by a variety of factors, including the severity of the loss, the nature of the loss, and many other factors across the auditory system that contribute to a unique profile of auditory function with hearing loss (see Table 2, Table 3 and Table 4). The addition of the capacity to integrate a measurement of the acoustic environment that hearing aids are being worn in [58] could be further combined with a machine learning approach to inform individual benefit targets. A deep neural network approach based on clinical datasets and research insights could help provide intelligent and personalised predictions of benefits for an individual with a hearing aid prescription (see Table 4). Ecological data logging [22] and evaluation [57] are increasingly accessible for use in clinical practice. With teleaudiology extending the flexibility of service delivery and with scientific learning continuing to develop, smarter approaches to validating fittings and supporting clinical decision-making could help us act sooner when low benefits occur, and provide better expectations, counselling, and additional support strategies when benefits are likely to be the most challenging (see Table 3).

**Table 4:** Insights into auditory processing and the impacts on trends and opportunities in clinical audiology.

Current Scientific Insights	Clinical Implications	Teleaudiology Applications
A single nerve cell collates the signals derived from multiple IHCs and OHCs before triggering a larger joint signal, which then travels up the nerve cells towards the next junction in the pathway. The characteristics of the combined signal are shaped by the contributing signals from the OHCs and IHCs, among other factors [59]. The implication of varying proportions of IHCs and OHCs before triggering a collective signal provides variety in the characteristics of any nerve signal, e.g., portraying intensity and frequency. Therefore, ‘minor’ auditory damage to the function of IHCs and OHCs that does not produce clinically measurable threshold changes may still be detected within the auditory system.	Tinnitus is an internally generated effect that is traditionally difficult to capture with objective measures. Current objective testing methods can, however, capture evidence of the related effects on speech recognition performance, i.e., above-average performance in quiet and below-average performance in noise, in the absence of changes in thresholds. Together, these data can indicate the presence of the compensatory amplification mechanisms that underlie issues such as tinnitus [17,60].	Subjective measurements, i.e., patient-reported experience/outcome measures (PREMs/PROMs), such as ecological momentary assessments (EMAs), can be captured via hearing-aid-supporting apps [18]. Teleaudiology-enabled EMAs can help complement clinically derived objective data [31] and, in turn, help support clinical decision-making for rehabilitation planning and monitoring.
Detection of changes in signalling patterns can lead to compensatory post-cochlea amplification mechanisms that may initially be helpful in improving discrimination, for example, of speech in low-level noise, but the same mechanisms can become excessive and burdensome in other situations [61,62]. For example, compensation may lead to the amplification of natural spontaneous cellular/neural activity or the excessive amplification of sounds that can make the perception of loudness distorted. The signalling pathway from the cochlea to the top of the neurological communication tree, i.e., the auditory cortex, contains multiple points where signalling can be triggered to travel back down the pathway (top-down processing) to modify the signalling activity through a positive or negative feedback mechanism. This feedback signalling mechanism can be referred to as ‘top-down processing’, i.e., feedback to OHCs to further amplify or dampen their mechanical activity in response to a soundwave being detected. The effects of aging can impact the efficacy and integrity of neurological signalling, including in feedback pathways, creating an immense variety of characteristic changes in an individual’s hearing loss.	Testing methods using competing sound (noise) can reveal differing auditory performance according to whether the test method relies on cognitively dependent speech content and the intensity (volume) of testing, i.e., near the threshold vs. comfortable audibility. The implication of the type of test being used is that the day-to-day difficulties that a person experiences may not be well represented in a clinical context depending on the chosen assessment strategy. Models of how loudness is identified and processed by the auditory system would benefit from incorporating new insights related to the type and nature of damage in the auditory system and the varying impacts at different loudness levels [63].	The growing array of testing methods that are available for clinical use with or without teleaudiology compatibility is increasing the potential volume of data to be collected, analysed, and used to inform clinical decision-making. Machine learning can aid in pattern recognition for the identification of predictive trends and key factors [42]. Deep neural networks can aid in building detailed maps for the increasingly complex and interconnected auditory system [26]. The continual addition of data and revision of parameters from research insights could lead to the identification of more effective ways to personalise audiological practices. The automation of data collection, teleaudiology-enabled improvements to clinical processes and workload planning, and teleaudiology-supported behavioural engagement with people wearing hearing aids can all contribute towards improving the success of audiological practices.

#### 2.2.2. Cochlear Implants

Both hearing aids and cochlear implants utilise microphones to sense the environment, similar but different processing methods to adapt sounds, and very different methods of stimulating the auditory pathway. Hearing aids deliver sound into the natural acoustic pathway (typically the ear canal), whereas cochlear implants use surgically implanted electrodes to electronically introduce an audio stimulus into the neural, i.e., post-cochlea, component of the auditory pathway (see Steps 4 and 9 in Table 2). The far more invasive surgical method of physically ‘fitting’ a cochlear implant means that the initial programming of the device occurs at a delayed time point after the surgery itself to allow time for healing. However, the connectivity of cochlear implants, as for hearing aids, has allowed remote ‘fitting’ (referred to as mapping for cochlear implants) to occur seamlessly via supporting apps, with no need for extra hardware since the surgical position has already been addressed [64,65]. Further considerations in the fitting process with cochlear implants include the healing of soft tissue following the surgery, which was also shown to be equitably triaged for referral using digital evaluation, thus further supporting clinical opportunities to consider teleaudiology-enabled choices in the planning and delivery of care for cochlear implant recipients [66].

The validation of the benefits from a cochlear implant is similar to that for hearing aids, with the objective of assuring that one’s hearing performance has been improved compared with that prior to the implant. Remote speech-based tests have been shown to have performance comparable to that of clinic-based procedures [67]. The capacity for clinicians to oversee the success of fitting outcomes with teleaudiology as the mode of service delivery has also shown encouraging results, supporting the notion that clinicians can utilise remote services to better triage and prioritise their workload without impacting clinical outcomes [68]. Combining the ecological capture of environmental data to inform the interpretation of any issues experienced would apply to cochlear implant recipients as it would to remote users of hearing aids.

### 2.3. Rehabilitation

The rehabilitative stage of clinical audiological practice commonly occurs as the post-assessment stage. When hearing aids have been prescribed, the rehabilitation stage is that which occurs after the fitting of hearing aids. When hearing aids are the key treatment, adherence to wearing them daily is the key objective for the desired benefits in hearing performance to be achieved. For adult recipients of cochlear implants, rehabilitation is focused on the acclimatisation of the person and their auditory system to a new experience of sound, whereby perception can initially be so different that speech is initially unintelligible. The importance of the rehabilitation period for a cochlear implant highlights the key idea that the intervention itself (the hearing aid or implant) is not the be-all and end-all of the potential benefit that a person could achieve. The basic neurological principle of “using it or losing it” underpinning audiological rehabilitation does not end with the fitting of a device [47,48]—whether a hearing aid or cochlear implant—for the same reasons that a hearing device cannot reverse permanent neurological damage and loss of function. Motivation for auditory training on top of fitting a hearing device is demonstrated by improved outcomes in auditory-processing-related skills and performance [69,70,71]. The complex nature of auditory processing and the overlap with cognitive function are also evident in the cognitive benefits observed with auditory training [70,71]. A key area of interest in audiological practice that is explicitly implicated in auditory processing is tinnitus (see Table 4). Auditory rehabilitation with tinnitus commonly centres on targeted cognitive and auditory processes and stimuli, which can be personalised and managed via teleaudiology [72,73]. Rehabilitation and auditory training programs for hearing aid users and cochlear implant recipients are already available via teleaudiology [70,71].

A key observation made by Nancy Tye-Murray in 2021 [20], emulating James Jerger’s comments in 1963 [6], is that the technology now at hand has an opportunity to outsource certain tasks, including specialist tasks, so that clinicians can potentially prioritise areas such as complex needs and those who cannot be assisted remotely, as a more efficient approach to managing their workload. An earlier study of online behavioural support for tinnitus also demonstrated a reduction in time needed by clinicians in comparison with group programs, with an estimated improvement in cost-effectiveness of 1.7 times [74]. Taken together, in the rehabilitation phase, teleaudiology has potential to help change the clinical workload and allocation of time for a smarter rather than harder choice of tasks.

Potential outcomes in auditory rehabilitation are a complex topic, further flawed by the fact that returning to ‘normal’ is not a feasible auditory objective [45]. Training schedules for auditory training programs have shown that extended durations or frequencies of training do not extend the potential improvement, indicating that a prediction of potential improvements made with auditory training would be multi-factorial and is not yet fully understood [75,76]. There have, however, been indicators that pre-training factors may be of interest for predicting and, therefore, planning whether to adopt auditory training as part of a rehabilitation program. Speech recognition and perception abilities have been noted to correlate with predicted improvements after training [69], and the mode of delivery (free-field listening vs. audio streamed via hearing aids) has been found to impact improvement scores [77]. Clinical adoption of new testing methods and the data they provide could be beneficial in eventually elucidating predictive factors and limitations to be considered in clinical decision-making with planning of auditory training programs. Interpretation of data pertaining to complex processes, such as auditory processing, can, however, also be impacted by new research-based insights that complicate or reveal additional complexity. Interpretation of speech testing data is now recognised to be impacted by the cognitive elements involved, becoming more complicated in the instance of the co-morbid occurrence of cognitive decline with hearing loss [78,79]. Speech-in-noise testing that does not rely on contextual factors, e.g., semantic or lexical content, is not as dependent on cognitive processing and can potentially indicate where specifically cochlea-based damage exists [16]. Language-independent testing methods can also provide versatility in excluding language-dependent cognitive processing or, more practically, the demographic challenge of non-native speakers in this increasingly globalised world [80]. Baseline speech performance in context-dependent and context-independent tests could support clinical decision-making in deciding whether to focus on auditory, cognitive, or combined training programs, where the same tests can help monitor progress or decide when to adapt or cease a given program. Training programs available via teleaudiology could allow clinicians to outsource work that can be performed remotely, allowing clinical decision-making to take priority in the clinical workload, as Nancy Tye-Murray and James Jerger may suggest [6,20].

Advancements in technology observed with deep neural networks and machine learning for improving assessment, auditory profiling, and, potentially, how a ‘best-match’ treatment can be selected could also be beneficial in clinical decision-making regarding auditory training programs. Utilising machine learning methods to identify what factors determine potential improvement as a product of diagnostic data and other individual factors may allow those who would require more complex help to continue to be seen at a clinic, while those who are likely to find success within currently available teleaudiology-enabled programs are able to do so. Ultimately, the specialised skills, knowledge, and training of audiology clinicians may be more efficient when tasks that can be satisfactorily managed with teleaudiology-compatible methods can be outsourced and the needs of the clinician that are best addressed in a clinic are prioritised.

## 3. Limitations

This narrative review sought to bring clarity to various medical underpinnings in new and emerging methods of teleaudiology and opportunities for clinical practice. Consequently, it was not possible to capture all avenues or discuss them all with adequate depth and contrast. The first limitation of this study is the limited number of topics that could be chosen, and more discussion is warranted. While this study could not address every relevant part, it is the expectation of the authors that enough discussion has been raised to encourage readers to pursue wider reading or to consider some of the topics raised with interest and willingness to continue the conversation. The growing understanding of the complex, integrated, and multi-layered process of auditory processing is an exciting area, touching on central auditory processing disorder, hyperacusis, and tinnitus, which all warrant more extensive reading and discussion.

The second key limitation of this review is the deliberate focus on the medical–technical underpinnings of teleaudiology methods. The field of teleaudiology has been in clinical practice for over a decade, with a wealth of available literature regarding clinical benefits, validation, and outcomes of various teleaudiology methods. The gap that this study addressed is the link from clinical practice to the newly informed medical–technical context. The reader may be provided with a holistic understanding of teleaudiology as a tool that they may wish to use in specific contexts and with specific reasoning. The future of teleaudiology is no longer one of being siloed; therefore, integration requires an integrative view of the field as a whole. The authors encourage the reader to complement the content of this study with the wider literature on teleaudiology available today for fair and reasonable clinical judgements in their own clinical practices.

The final limitation is a conscious restriction of the scope of the review to adult audiological practice. While many of the clinical methods may be equally relevant to younger persons requiring audiological care, there is an inherent difference in the autonomy that can be afforded, decision-making processes, as well as possible variance in the developmental relevance of the functional processes of the younger auditory system. The authors, therefore, focused on the adult context, whereby any insights and learnings would be encouraged to be verified rather than directly applied to the clinical practices of young persons.

## 4. Future Directions

Research and innovation are continually expanding the information and opportunities available in any field of clinical medicine. While scientific insights and the technologies available in a clinic may not always emerge in line with each other, the gap between what we know and what we can do should always be challenged to enable improvements in possible and potential clinical practices and success thereof. The emergence of teleaudiology has provided opportunities to challenge the barriers of logistics, access, and, potentially, engagement in the provision of audiological care. Adoption of and adherence to hearing aids have been the largest barriers to successful management of hearing loss, with evidence supporting the versatility in how teleaudiology can be used in various ways to improve adoption and adherence. Overall, making better use of the tools available today is akin to the automation of the audiometer 60 years ago, which allowed clinicians to be smarter rather than being stuck working harder to conduct the work that a trained and qualified professional is able to perform. Audiology may just be at the stage of progress in its journey as a field of healthcare where great increases in access, methodologies, treatment activities, and clinical tools to aid decision-making are occurring. Audiology clinicians may come to gain a significantly wider array of automated and smart clinical tools, providing an opportunity to efficiently and effectively improve the success of clinical audiology practices.

## Figures and Tables

**Table 1:** Clinical practice guidance in adult services from Australia, the USA, and the UK ^1^.

Clinical Guidelines:	Assessment	Fitting of Devices	Rehabilitation
Audiology Australia: Professional Practice Guidelines [2]	Domain 4: identifying ear and hearing conditions	Domain 5: rehabilitation support 5.6–8 technological aids	Domain 5: rehabilitation support 5.1–5.4 and 5.14
Audiology Australia: Australian Teleaudiology Guidelines [3]	Clinical guidance: hearing screening and audiological assessment	Hearing and assistive devices—fittings, adjustment, and aftercare	Hearing and assistive devices—fittings, adjustment, and aftercare
American Academy of Audiology: Standards of Practice for Audiology [4]	Standard II identification (screening) and Standard III evaluation/(diagnosis)	Standard IV Treatment A. 1	Standard IV Treatment A. 2
British Society of Audiology: Various Guidance Documents [5]	Tympanometry and acoustic reflex thresholds, pure tone air and bone conduction threshold audiometry with and without masking, and assessment of speech understanding in noise in adults with hearing difficulties	BAA BSA Remote Fitting Guidance	Adult rehabilitation—common principles in audiology services

^1^ All guidelines and standards cited are for adult audiological practice.

**Table 2:** Steps and stages of the audio pathway, from the environmental acoustic origin to the completion of auditory processing. Mapping of the various processes requires a scaffold to align the composite parts.

Location	Stage
(1) Sound sources	Environmental
(2) Soundwaves travel through air	Environmental
(3) Outer ear (pinna)	Conductive
(4) Outer ear (ear canal and ear drum)	Conductive
(5) Middle ear	Conductive
(6) Inner ear (basilar membrane)	Conductive
(7) Inner ear (hair cells)	Sensory
(8) Inner ear (nerve cells)	Neural
(9) Inner ear (shared neurons)	Neural
(10) Brain (temporal lobe)	Auditory processing
(11) Brain (cognitive complexity)	Auditory processing
